# The Use of Hexokinase 2-Displacing Peptides as an Anti-Neoplastic Approach for Malignant Peripheral Nerve Sheath Tumors

**DOI:** 10.3390/cells13131162

**Published:** 2024-07-08

**Authors:** Francesco Ciscato, Ionica Masgras, Alessandro Gori, Marco Fantuz, Greta Bergamaschi, Denis Komarov, Martina La Spina, Shiva Ghasemi-Firouzabadi, Marco Pizzi, Angelo Paolo Dei Tos, Federica Chiara, Alessandro Carrer, Andrea Rasola

**Affiliations:** 1 Department of Biomedical Sciences, University of Padova, 35131 Padova, Italy; 2 Institute of Neuroscience, National Research Council (CNR), 35131 Padova, Italy; 3 SCITEC Institute of Chemical Science and Technology “Giulio Natta”, National Research Council (CNR), 20133 Milano, Italy; 4 Veneto Institute for Molecular Medicine (VIMM), 35129 Padova, Italy; 5 Department of Biology, University of Padova, 35131 Padova, Italy; 6 General Pathology and Cytopathology Unit, Department of Medicine (DMED), University of Padova, 35128 Padova, Italy; 7 Department of Surgery, Oncology and Gastroenterology, University of Padova, 35128 Padova, Italy; 8 Veneto Institute of Oncology IOV-IRCCS, 35128 Padova, Italy

**Keywords:** malignant peripheral nerve sheath tumors, hexokinase 2, hexokinase 2 targeting peptides, Neurofibromatosis type 1

## Abstract

Malignant Peripheral Nerve Sheath Tumors (MPNSTs) are aggressive sarcomas that can arise both sporadically and in patients with the genetic syndrome Neurofibromatosis type 1 (NF1). Prognosis is dismal, as large dimensions, risk of relapse, and anatomical localization make surgery poorly effective, and no therapy is known. Hence, the identification of MPNST molecular features that could be hit in an efficient and selective way is mandatory to envision treatment options. Here, we find that MPNSTs express high levels of the glycolytic enzyme Hexokinase 2 (HK2), which is known to shield cancer cells from noxious stimuli when it localizes at MAMs (mitochondria-associated membranes), contact sites between mitochondria and endoplasmic reticulum. A HK2-targeting peptide that dislodges HK2 from MAMs rapidly induces a massive death of MPNST cells. After identifying different matrix metalloproteases (MMPs) expressed in the MPNST microenvironment, we have designed HK2-targeting peptide variants that harbor cleavage sites for these MMPs, making such peptides activatable in the proximity of cancer cells. We find that the peptide carrying the MMP2/9 cleavage site is the most effective, both in inhibiting the in vitro tumorigenicity of MPNST cells and in hampering their growth in mice. Our data indicate that detaching HK2 from MAMs could pave the way for a novel anti-MPNST therapeutic strategy, which could be flexibly adapted to the protease expression features of the tumor microenvironment.

## 1. Introduction

Malignant peripheral nerve sheath tumor (MPNST) is an aggressive soft-tissue sarcoma of peripheral nerves, whose 5-year overall survival is approximately 20 to 50%. Malignant transformation can occur either sporadically or in patients with the tumor-predisposing genetic syndrome Neurofibromatosis type 1 (NF1), caused by loss of function mutations in the tumor suppressor NF1 gene encoding the Ras-GTPase activating protein (Ras-GAP) neurofibromin [1]. In NF1 patients, MPNSTs evolve from benign plexiform neurofibromas (PNs) elicited by loss of heterozygosity at the NF1 locus in Schwann cells (SCs) or in pluripotent cells of the neural crest [2]. MPNSTs are characterized by a high number of genetic alterations, including the loss of CDKN2A or TP53 and the inactivation of polycomb repressive complex 2 (PRC2), which lead to extensive changes in histone methylation landscape, somatic copy number aberrations, chromosomal losses or amplification, and even whole genome doubling [3]. This high degree of genomic instability sustains the phenotypic variability of MPNST, and recent multiomic analyses have provided further insights into its molecular heterogeneity by unveiling the presence of several subpopulations of malignant cells in each tumor mass [4].

Current treatments are limited to surgical resection, which is rarely effective and not always feasible due to the large size of the tumor or its localization, combined with adjuvant anthracycline-based chemotherapy or radiotherapy, but the overall clinical benefit remains poor [5]. Moreover, MPNSTs have the tendency to relapse following therapy and are characterized by a high propensity for spreading and metastasizing. Taken together, these features make MPNSTs extremely challenging sarcomas to treat, and in NF1 patients, they constitute the first cause of death.

An overlooked aspect of MPNST biology is represented by the profound metabolic changes that must occur to sustain its growth and progression. We have shown that NF1-related tumor cells markedly enhance their glycolytic activity [6], and a marked rise in glucose avidity is recorded by positron emission tomography when benign PNs progress toward malignancy [7]. Even though the rewiring of MPNST metabolic circuits remains largely unknown, singling out some of its crucial components could disclose the unexplored perspective of identifying actionable vulnerabilities.

Hyperactivation of the glycolytic process requires a marked induction of Hexokinase 2 (HK2), the most efficient HK isozyme, which catalyzes the phosphorylation of glucose to make it available for intracellular utilization [8]. Here, we propose an innovative antineoplastic approach for hampering MPNST growth that is based on targeting HK2. We had previously found that HK2 displays antiapoptotic properties upon its binding to interfacing domains between endoplasmic reticulum and mitochondria called MAMs (mitochondria-associated membranes) [9,10]. As a consequence of its dual metabolic and antiapoptotic function, HK2 (over)expression is associated with unfavorable prognosis and radiotherapy/chemotherapy resistance in diverse tumor settings [8], whereas its silencing or genetic ablation abrogates neoplastic growth and restores sensitivity to antitumor treatments [11]. Several lines of evidence suggest that HK2 inhibition could be a strategy to counter MPNST growth. HK2 expression is known to be induced downstream to RAS signaling [11] and in a HIF-1-dependent way [12], whereas AKT-dependent phosphorylation is required for HK2 mitochondrial localization and its pro-survival activity [8]. In MPNST, functional loss of the Ras-GAP neurofibromin constitutively induces Ras signaling and Akt activity, and HIF-1-mediated transcription is enhanced [13,14]. Altogether these data strongly suggest an induction of HK2 expression, and indeed we have found that HK2 is present in the MAMs of MPNST cells [10].

These results set the stage for HK2 targeting in MPNST, but the use of pharmacological HK2 inhibitors has proven arduous, as side effects on other HK isozymes lead to systemic dysregulation of glucose metabolism and toxicity [15]. To overcome these problems, we have used a cell-penetrating peptide called HK2pep that displaces HK2 from MAMs without targeting its catalytic activity, with the aim of eliciting tumor cell apoptosis while avoiding off-target effects caused by metabolic disruptions. HK2pep has a modular structure composed of (1) the active sequence containing the N-terminal tail of HK2; (2) a polycation stretch, which allows HK2pep cell entrance and is shielded by (3) a polyanion stretch to prevent nonspecific cell uptake; and (4) a matrix metalloproteinase (MMP) cleavable sequence for the release of the active moiety. Upon MMP activation and cell entry, the active moiety of HK2pep displaces HK2 from MAMs, leading to a massive Ca^2+^ flux into mitochondria and to the ensuing tumor cell death, without off-target effects [10]. 

Here, after assessing the expression of HK2 in a panel of MPNST samples and cells, we have conceived an HK2-targeting strategy that takes into account the heterogeneous features of MPNSTs.

MMPs are highly expressed by many tumor types and contribute to neoplastic progression, but the MMP subtypes that characterize the microenvironment can vary, depending on tumor type and stage [16]. We have designed a set of HK2-targeting peptides cleavable by different MMPs that we found to be expressed in the MPNST microenvironment, selecting the molecule with the highest efficacy on MPNST cells, both in vitro and in vivo.

## 2. Materials and Methods

### 2.1. MPNST Cell Models

Human MPNST S462 cells were kindly provided by Dr. Conxi Lazaro (Institut Català d’Oncologia, Barcelona, Spain). Murine sMPNST and cisMPNST cells were kindly provided by Dr. Lu Q. Le (University of Texas Southwestern Medical Center, Dallas, TX, USA). Cells were grown in Dulbecco’s modified Eagle’s medium (DMEM) supplemented with 10% FBS, 1% glutamine, 1% sodium pyruvate, 100 µg/mL penicillin, and 100 µg/mL streptomycin at 37 °C in a 5% CO_2_ humidified atmosphere. 

### 2.2. Immunohistochemical Analysis

Histological analyses were performed on primary human MPNST samples (n = 5). In detail, 4 µm thick tissue sections were obtained from formalin-fixed paraffin-embedded tissue samples, and representative tumor areas were selected on H&E-stained slides for immunohistochemical (IHC) analysis. IHC was performed using the primary anti-HK2 antibody (sc-6521, Santa Cruz Biotechnology, Dallas, TX, USA). Antigen retrieval was performed with heat/EDTA in a Bond-Max automated immunostainer (Leica Biosystems, Milan, Italy).

### 2.3. Dataset Analyses

Publicly available datasets were obtained through data mining of the GEO Expression Omnibus database (https://www.ncbi.nlm.nih.gov/geo/ accessed on 30 September 2023). The entirety of the published datasets containing transcriptomic profiling of MPNST samples was obtained, including the accession numbers GSE14038, GSE17118, GSE35852, GSE52252, GSE66743, and GSE178989. For each dataset, only MPNST and PN samples were utilized for further analysis. To evaluate gene expression of HK2 and of the described panel of MMPs, the following analyses were performed: For microarray datasets (all of the mentioned ones aside GSE178989), expression was analyzed using the limma pipeline (DOI: 10.18129/B9.bioc.limma). Expressed genes were considered as having −0.9 z-scores of expression (normalized on gene expression in the full dataset, comprising both MPNST and PN samples). Of note, most (~90%) of the negative expression samples displayed undetectable expression levels for the selected genes; for the RNA-seq dataset GSE178989, the matrix of raw counts was obtained from the accession page, and negative expression samples were considered as the ones having zero counts for the selected genes.

### 2.4. Peptide Synthesis

Peptides were synthesized by microwave-assisted solid-phase procedures using a Biotage Alstra peptide synthesizer on a preloaded HMPB-resin (100–200 mesh-Iris Biotech GmbH, Bengaluru, India). The fluoren-9-ylmethoxycarbonyl (Fmoc) strategy was used throughout the peptide chain assembly as previously described [10]. Peptides were purified by a preparative reverse phase HPLC. Molecular masses of the peptide were confirmed by mass spectroscopy on a MALDI TOF-TOF using an Applied Biosystems 4800 mass spectrometer (Waltham, MA, USA). The purity of the peptides was about 95% as evaluated by analytical reverse-phase HPLC. MMP cleavable sequences were designed according to [17]. Peptides sequences reporting (if applicable) the MMP selectivity Z Score (from −2 to 3) for each cleavable site:

cl-SCRp: VGAHAGEYGAEALERRRRRRRRRRPLG;
cl-HK2p: MIASHLLAYFFTELNRRRRRRRRRPLG;
SCRpep: VGAHAGEYGAEALERRRRRRRRRRPLGLAG-Ahx-EEEEEEEE;
HK2pep_1: MIASHLLAYFFTELNRRRRRRRRRPLGLAG-Ahx-EEEEEEEE cleavable by MMP 2 and 9;
HK2pep_2: MIASHLLAYFFTELNRRRRRRRRRPHWLNAG-Ahx-EEEEEEEE cleavable by MMP 1 (Z Score 2.5) and 2 (Z Score 2.5);
HK2pep_3: MIASHLLAYFFTELNRRRRRRRRRPFMILQL-Ahx-EEEEEEEE cleavable by MMP 9 (Z Score 3);
HK2pep_4: MIASHLLAYFFTELNRRRRRRRRRPKGVVDM-Ahx-EEEEEEEE cleavable by MMP 13 (Z Score 2.5) and 14(Z Score 2.5).

### 2.5. Cell Viability Assays

Cell viability was assessed by cytofluorimetry on S462 and sMPNST cells. Cytofluorimetric recordings of phosphatidylserine exposure on the cell surface (increased staining of FITC-conjugated Annexin V; Roche, Basel, Switzerland) and loss of plasma membrane integrity (7-AAD staining; Sigma, Kawasaki, Japan) were repeated every 15 min on a BD LSRFortessa™ Cell Analyzer instrument (BD Biosciences, Franklin Lakes, NJ, USA) after treatment with cl-HK2p. Data were analyzed with FACSDiva software, v. 6.1.3. Double-negative cells were considered viable. 

The MTS CellTiter 96^®^ AQueous One Solution Cell Proliferation Assay was used to assess sMPNST and cisMPNST cell viability after treatment with cl-HK2p or HK2pep and according to the manufacturer’s instructions. The MMP inhibitor GM-6001 (Enzo Life Sciences, Farmingdale, NY, USA) was preincubated for 4 h. 

### 2.6. Western Immunoblot Analyses

Protein extraction of sMPNST-derived tumor samples and cell lysis of S462, sMPNST, and cisMPNST cells was performed in RIPA buffer (50 mM Tris-HCl, pH 7.4, 150 mM NaCl, 0.25% deoxycholic acid, 1% NP-40, 1 mM EDTA) supplemented with phosphatase and protease inhibitors (Sigma). Lysates were cleared by centrifugation at 18,000× *g* for 30 min at 4 °C. Protein quantification was performed with BCA Protein Assay Kit (Thermo Scientific-Pierce, Rockford, IL, USA). After SDS/PAGE gel electrophoresis, proteins were transferred onto nitrocellulose Hybond-C Extra membranes (Cytiva, Amersham, UK) and immunostained with anti-HK2 (H.738.7, Thermo Scientific, Waltham, MA, USA), anti-MMP1 (PA5-16498, ThermoFisher, Waltham, MA, USA), anti-MMP2 (ab92536, Abcam, Cambridge, UK), anti-MMP9 (ab137867, Abcam, Cambridge, UK), MMP13 (MA5-14238, ThermoFisher), MMP14 (PA5-13183, ThermoFisher), MCU (HPA016480, Merk, Rahway, NJ, USA) antibodies. Proteins were visualized utilizing the Odyssey Clx (Li-Cor, Lincoln, NE, USA) system according to manufacturer indications.

### 2.7. Focus Forming Assays

Cells were seeded in 12-well culture plates in standard DMEM medium with 10% FBS. When cells reached confluence, medium was replaced with DMEM containing 1% FBS (day 0). Treatments with peptides were performed after foci formation at day 5. Ten days after treatments (day 15), plates were washed in PBS, fixed in methanol for 30 min, and stained with GIEMSA solution for 1 h. Images were acquired using a Leica DMIL LED microscope equipped with a Leica ICC50 HD camera and analyzed with ImageJ software 1.47b.

### 2.8. Spheroid Formation Assays

Cells were seeded in low-adhesion 96-well culture plates with round bottoms and centrifuged at 400× *g* for 1 min to aggregate cells. Spheroids were kept in a standard DMEM medium with 1% FBS and after 3-day treatments with peptides were performed. Images were acquired using a Leica DMIL LED microscope equipped with a Leica ICC50 HD camera and analyzed with ImageJ software.

### 2.9. In Vivo Tumorigenesis Assays

Nude female mice (Charles River, Wilmington, MA, USA) were housed on a 12:12 h light/dark cycle at 25 °C in accordance with the European Community guidelines. Six-week-old animals (n ≥ 7) were injected in the left flank with 1.5 × 10^6^ sMPNST cells in 100 μL of serum-free sterile DMEM mixed with 4% Matrigel. Tumors were visible under the skin after 6 days when they reached the volume of 15 mm^3^ ca. At this stage, 40 nmol of HK2pep was intraperitoneally administered twice a day for 6 days when mice were sacrificed. Ultrasound inspections were performed using Vevo 2100 (Visualsonic, Fujifilm, Toronto, ON, Canada) every 2 days. Measurements, 3D reconstructions of tumors, and volume calculations were performed using VevoLAB 1.7.0.7071 software. Italian Ministry permission number: 286/2021-PR.

## 3. Results

### 3.1. HK2 Expression in MPNST Cells Makes Them Vulnerable to HK2pep 

**Figure 1:**
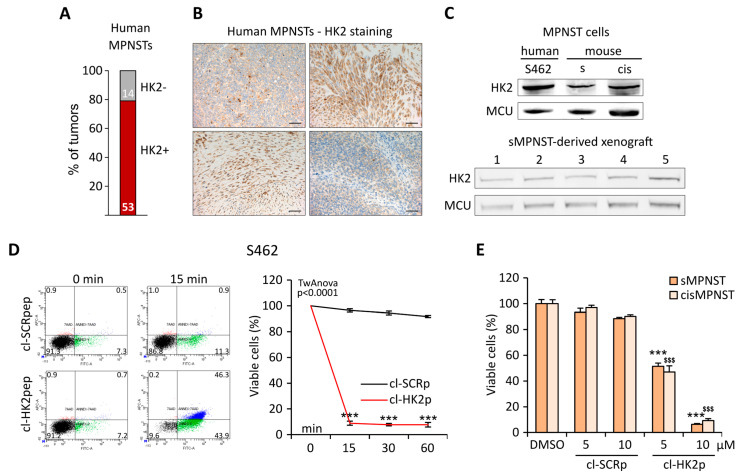
HK2 expression analysis and cell death induction by cl-HK2p in MPNST cells: (**A**) Stacked bar plot displaying the percentage of tumors expressing the HK2 transcript from a panel of 67 MPNST samples derived from publicly available datasets (described in the Methods section). Numbers in the bars display the absolute count of samples in each subset. (**B**) Representative immunohistochemistry analyses of HK2 expression in human MPNST samples (scale bar 200 µm). (**C**) Western blot analysis of HK2 expression in human (S462) and murine (sMPNST, cisMPNST) MPNST cell models and in sMPNST-derived xenograft tumor samples; MCU (mitochondrial calcium uniporter) was used as a loading control. (**D**,**E**) Cell viability assessed either by cytofluorimetry on S462 cells treated with 5 µM peptides (**D**); viable cells are double negative for Annexin V-FITC and 7-AAD staining) or by an endpoint MTS analysis on sMPNST and cisMPNST cells after 60 min of treatment with 5 µM or 10 µM peptides (**E**). cl-HK2p: cleaved HK2-targeting peptide; cl-SCRp: cleaved scrambled peptide; (**D**) two-way ANOVA *p* < 0.0001, Student’s *t*-test followed by Bonferroni post hoc test *** *p* < 0.001; (**E**) Student’s *t*-test *** *p* < 0.001, ^$$$^ *p* < 0.001.

We used different approaches to analyze the presence of HK2 in MPNST samples. Mining publicly available datasets revealed a high level of HK2 mRNA in 53 out of 67 (80%) human patients (Figure 1A); accordingly, the HK2 protein is strongly expressed in a set of human samples (Figure 1B), in human and mouse MPNST cell models, and in tumors derived from xenografting MPNST cells in nude mice (Figure 1C). In order to test the efficacy of our HK2-displacing strategy, we treated MPNST cells with the active portion of HK2pep (cleaved HK2pep, cl-HK2p), composed of the HK2-targeting sequence and the cell-penetrating polycation moiety but lacking the MMP-cleavable portion and the polyanion shielding stretch. Both human and murine MPNST cells massively underwent apoptosis after a few minutes of cl-HK2p administration (Figure 1D,E; Supplementary Figure S1A). The use of a MMP inhibitor demonstrates that administration of the activatable HK2pep decreases MPNST cell viability in a MMP-dependent manner (Supplementary Figure S1B).

### 3.2. Analysis of MMP Expression in MPNST Models

**Figure 2:**
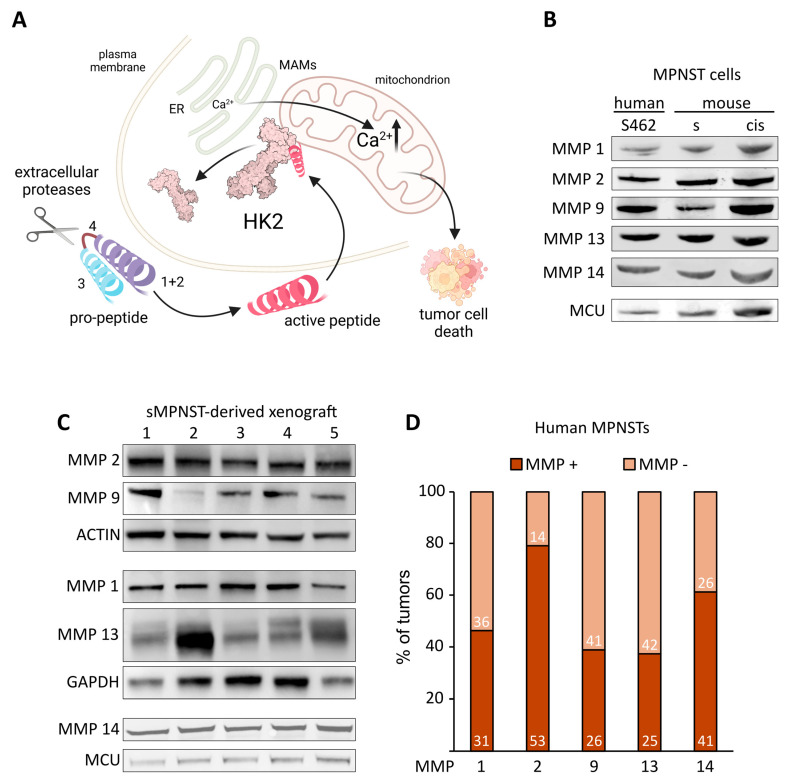
MMP expression in MPNST models: (**A**) HK2pep mode of action. After activation by extracellular matrix metalloproteases (MMPs), the active, cleaved form of the peptide (cl-HK2p) enters the target cell and displaces HK2 from MAMs, causing a massive flux of Ca^2+^ into mitochondria and the ensuing cell death; 1, 2, 3, and 4 represent the different modules that compose HK2pep (see main text); created with BioRender.com. (**B**,**C**) Western immunoblot analysis of MMP-1, MMP-2, MMP-9, MMP-13, and MMP-14 expression in S462, sMPNST, cisMPNST cells (**B**) and in sMPNST-derived xenograft tumors (**C**); MCU, GAPDH or actin were used as loading controls. (**D**) Stacked bar plot displaying the percentage of tumors expressing the transcripts of MMP-1, MMP-2, MMP-9, MMP-13, and MMP-14 from a panel of 67 MPNST samples derived from publicly available datasets. Numbers in the bars display the absolute count of samples in each subset.

HK2pep sequences can be designed to contain different MMP-cleavable motifs (module 4 in Figure 2A). Matching such motifs with the proteases that are present in a specific tumor microenvironment opens the possibility of adapting the HK2-displacement approach to several tumor types. 

Previous studies have found that MMP1, MMP2, and MMP13 are expressed in some MPNST samples [18,19]. We extended these analyses, finding the expression of MMP-1, MMP-2, MMP-9, MMP-13, and MMP-14 in MPNST cell models, as well as in MPNST cell-derived xenografts (Figure 2B,C). Consistently, a meta-analysis carried out on 67 human MPNSTs confirmed the expression of the same group of MMPs in a high proportion of samples (Figure 2D). 

### 3.3. Tailoring the HK2 Displacement Approach to MPNSTs

**Figure 3:**
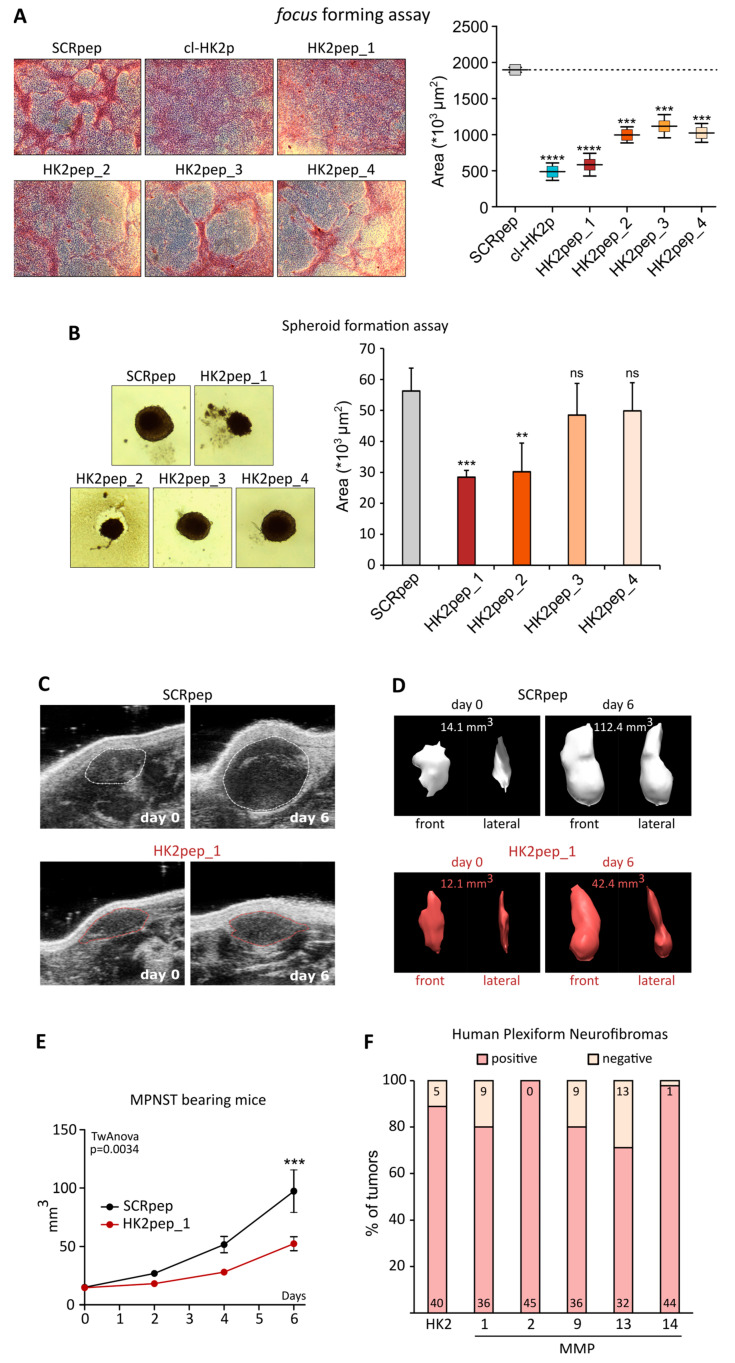
Tailored HK2peps efficacy in tumorigenic condition: (**A**) Focus forming assay on sMPNST cells; representative pictures of foci (**left**) and focus area quantification (**right**) after 10 days of 2 µM HK2peps treatment are shown. (**B**) Spheroid formation assay on sMPNST cells; representative pictures (**left**) and spheroid area quantification (**right**) are shown; 2 μM HK2peps treatments were applied for 12 days renewing half media and treatments every 3 days. Data are reported as mean±SEM values; ** *p* < 0.01, *** *p* < 0.001, **** *p* < 0.0001, *ns* = non-significant, with a Student’s *t*-test. (**C**–**E**) Analyses of sMPNST-derived xenograft tumors treated with either SCRpep or HK2pep_1 (40 nmol/injection twice a day for 6 days; intraperitoneal administration). Peptide administration started at day 0 in the Figure, i.e., 6 days after MPNST cell injection under the skin. In (**C**), representative ultrasound inspections of the tumors (tumors are highlighted by deashed lines); in (**D**), ultrasound-based 3D reconstructions and volume calculations of the tumors; in (**E**), tumor volume growth quantification. Data are reported as mean ± SEM with *p* = 0.0034 with a two-way ANOVA test, *** *p* < 0.001 with a Student’s *t*-test followed by Bonferroni post-hoc test. (**F**) Stacked bar plot displaying the percentage of tumors expressing the transcripts of HK2, MMP-1, MMP-2, MMP-9, MMP-13, and MMP-14 from a panel of 45 plexiform neurofibroma samples derived from publicly available datasets. Numbers in the bars display the absolute count of samples in each subset.

Building on the MMP expression pattern in MPNSTs, we designed and synthesized a set of HK2peps carrying sequences cleavable by MMP-2 and MMP-9 (HK2pep_1), MMP-1 and MMP-2 (HK2pep_2), MMP-9 (HK2pep_3), or MMP-13 and MMP-14 (HK2pep_4). In order to compare the relative efficacy of the HK2pep variants in inhibiting MPNST cell tumorigenicity, we started with in vitro focus forming and spheroid formation assays. All peptides decreased the formation of foci, and the peptide carrying the MMP2/9 cleavable sequence (HK2pep_1) showed the most effective result (Figure 3A). The formation of MPNST cell spheroids was hampered both by HK2pep_1 and by the peptide carrying MMP1/2 cleavable sequences (HK2pep_2), whereas the other peptide variants were not effective (Figure 3B). Following the results of these in vitro tumorigenic assays, we decided to use HK2pep_1 for in vivo treatment on MPNST cells grafted in the flank of nude mice. When tumors became visible, 40 nmol of HK2pep_1 was intraperitoneally administered twice a day for 6 days. Echograph inspections, 3D tumor reconstructions, and growth curves (Figure 3C–E) demonstrated that HK2pep_1 could markedly reduce the growth of MPNST cell-derived tumor masses.

## 4. Discussion

Our study identifies HK2 as a protein that can constitute the target for an innovative anti-neoplastic approach in MPNSTs. Displacing HK2 from MAMs induces massive death of MPNST cells in the range of minutes. HK2 is expressed in diverse cancer cell types, where it mainly localizes in MAMs, whereas it is absent in most non-tumor cells. 

Importantly, HK2peps are impermeable across endothelia but can cross the fenestrated vessels that characterize the tumor microenvironment, where MMPs are strongly induced. Even if local MMP activation, as in the case of wound healing or inflammation processes, could in principle release HK2peps in non-tumor environments, most non-neoplastic cells do not express the isoform 2 of the hexokinase, and when it is expressed, it barely interacts with MAMs. HK2peps can also traverse glomerular endothelium to be excreted, further diminishing the risk of side effects caused by a prolonged presence in the blood flow. Taken together, these features minimize any possible off-target toxic effect of the HK2-targeting peptides [10]. The use of HK2peps characterized by different MMP cleavage sequences demonstrates that this approach is flexible and adaptable to the specific features of each MPNST. In principle, a thorough characterization of the proteases actually present in the MPNST extracellular environment at different neoplastic stages could allow the optimization of the design of the most effective HK2peps and match their choice with dynamic changes in the microenvironment MMP composition that could occur during MPNST evolution. As a perspective, a combination of different HK2peps could be the ideal way to efficiently deliver their active moiety, thus boosting their overall anti-neoplastic effect. 

It is also worth considering that while approximately half of the MPNSTs are sporadic, half arise in NF1 patients from benign PNs [7]. We analyzed public datasets, finding a high expression level of both HK2 and MMP-1/2/9/13/14 in benign PNs of NF1 patients (Figure 3F), disclosing the possibility of starting treatments with HK2peps before these tumors switch to the malignant phenotype. Further experiments will aim at evaluating the efficacy of this approach in genetic mouse models that closely recapitulate the onset and growth of MPNSTs, also taking into account the role played by the peripheral nerve microenvironment. 

Finally, it must be highlighted the possibility of using HK2peps in advanced or relapsing cancer stages, as HK2 induction characterizes chemoresistance in several tumor models and HK2 targeting in tumor cells sensitize them to chemo/radiotherapy.

## 5. Conclusions

Our data suggest that the use of HK2-displacing peptides, alone or in association with other anti-neoplastic approaches, could represent a starting point for the development of future personalized treatments for MPNST and possibly other aggressive malignancies. 

## Data Availability

The original contributions presented in the study are included in the article/Supplementary Material, further inquiries can be directed to the corresponding author/s.

## Supplementary Materials

The following supporting information can be downloaded at https://www.mdpi.com/article/10.3390/cells13131162/s1, Figure S1: (**A**) Cell death assessed by cytofluorimetry on sMPNST cells treated with 5 μM peptides (viable cells are double negative for Annexin V-FITC and 7-AAD staining). (**B**) Cell viability assessed by endpoint MTS analysis on sMPNST cells after 60 min of treatment with 10 μM HK2pep; the MMPs inhibitor GM-6001 was preincubated for 4 h. cl-HK2p: cleaved HK2-targeting pep-tide; cl-SCRp: cleaved scrambled peptide; (**A**) two-way ANOVA *p* < 0.0001, Student’s *t*-test followed by Bonferroni post-hoc test *** *p* < 0.001; (**B**) Student’s *t*-test *** *p* < 0.001.

## References

[1] Reilly K.M., Kim A., Blakely J., Ferner R.E., Gutmann D.H., Legius E., Miettinen M.M., Randall R.L., Ratner N., Jumbé N.L. (2017). Neurofibromatosis Type 1—Associated MPNST State of the Science: Outlining a Research Agenda for the Future. J. Natl. Cancer Inst..

[2] Jiang C., McKay R.M., Le L.Q. (2021). Tumorigenesis in neurofibromatosis type 1: Role of the microenvironment. Oncogene.

[3] Cortes-Ciriano I., Steele C.D., Piculell K., Al-Ibraheemi A., Eulo V., Bui M.M., Chatzipli A., Dickson B.C., Borcherding D.C., Feber A. (2023). Genomic Patterns of Malignant Peripheral Nerve Sheath Tumor (MPNST) Evolution Correlate with Clinical Outcome and Are Detectable in Cell-Free DNA. Cancer Discov..

[4] Wu L.M.N., Zhang F., Rao R., Adam M., Pollard K., Szabo S., Liu X., Belcher K.A., Luo Z., Ogurek S. (2022). Single-cell multiomics identifies clinically relevant mesenchymal stem-like cells and key regulators for MPNST malignancy. Sci. Adv..

[5] Somatilaka B.N., Sadek A., McKay R.M., Le L.Q. (2022). Malignant peripheral nerve sheath tumor: Models, biology, and translation. Oncogene.

[6] Masgras I., Ciscato F., Brunati A.M., Tibaldi E., Indraccolo S., Curtarello M., Chiara F., Cannino G., Papaleo E., Lambrughi M. (2017). Absence of Neurofibromin Induces an Oncogenic Metabolic Switch via Mitochondrial ERK-Mediated Phosphorylation of the Chaperone TRAP1. Cell Rep..

[7] González-Muñoz T., Kim A., Ratner N., Peinado H. (2022). The Need for New Treatments Targeting MPNST: The Potential of Strategies Combining MEK Inhibitors with Antiangiogenic Agents. Clin. Cancer Res. Off. J. Am. Assoc. Cancer Res..

[8] Ciscato F., Ferrone L., Masgras I., Laquatra C., Rasola A. (2021). Hexokinase 2 in Cancer: A Prima Donna Playing Multiple Characters. Int. J. Mol. Sci..

[9] Chiara F., Castellaro D., Marin O., Petronilli V., Brusilow W.S., Juhaszova M., Sollott S.J., Forte M., Bernardi P., Rasola A. (2008). Hexokinase II detachment from mitochondria triggers apoptosis through the permeability transition pore independent of voltage-dependent anion channels. PLoS ONE.

[10] Ciscato F., Filadi R., Masgras I., Pizzi M., Marin O., Damiano N., Pizzo P., Gori A., Frezzato F., Chiara F. (2020). Hexokinase 2 displacement from mitochondria-associated membranes prompts Ca^2+^-dependent death of cancer cells. EMBO Rep..

[11] Patra K.C., Wang Q., Bhaskar P.T., Miller L., Wang Z., Wheaton W., Chandel N., Laakso M., Muller W.J., Allen E.L. (2013). Hexokinase 2 is required for tumor initiation and maintenance and its systemic deletion is therapeutic in mouse models of cancer. Cancer Cell.

[12] Schito L., Rey S. (2018). Cell-Autonomous Metabolic Reprogramming in Hypoxia. Trends Cell Biol..

[13] Ratner N., Miller S.J. (2015). A RASopathy gene commonly mutated in cancer: The neurofibromatosis type 1 tumour suppressor. Nat. Rev. Cancer.

[14] Rad E., Dodd K., Thomas L., Upadhyaya M., Tee A. (2015). STAT3 and HIF1α Signaling Drives Oncogenic Cellular Phenotypes in Malignant Peripheral Nerve Sheath Tumors. Mol. Cancer Res. MCR.

[15] Fortunato S., Bononi G., Granchi C., Minutolo F. (2018). An Update on Patents Covering Agents That Interfere with the Cancer Glycolytic Cascade. ChemMedChem.

[16] Kessenbrock K., Plaks V., Werb Z. (2010). Matrix metalloproteinases: Regulators of the tumor microenvironment. Cell.

[17] Kukreja M., Shiryaev S.A., Cieplak P., Muranaka N., Routenberg D.A., Chernov A.V., Kumar S., Remacle A.G., Smith J.W., Kozlov I.A. (2015). High-Throughput Multiplexed Peptide-Centric Profiling Illustrates Both Substrate Cleavage Redundancy and Specificity in the MMP Family. Chem. Biol..

[18] Nabeshima K., Iwasaki H., Nishio J., Koga K., Shishime M., Kikuchi M. (2006). Expression of emmprin and matrix metalloproteinases (MMPs) in peripheral nerve sheath tumors: Emmprin and membrane-type (MT)1-MMP expressions are associated with malignant potential. Anticancer Res..

[19] Holtkamp N., Atallah I., Okuducu A.-F., Mucha J., Hartmann C., Mautner V.-F., Friedrich R.E., Mawrin C., von Deimling A. (2007). MMP-13, p53 in the progression of malignant peripheral nerve sheath tumors. Neoplasia.

