# Supplementary material for: The Use of Hexokinase 2-Displacing Peptides as an Anti-Neoplastic Approach for Malignant Peripheral Nerve Sheath Tumors
Source: Cells. 2024 Jul 8;13(13):1162. doi: 10.3390/cells13131162 (PMC11240344; doi:10.3390/cells13131162)
Supplement: Supplementary file 1 [file cells-13-01162-s001.zip › cells-3028709-supplementary.pdf]

cells

MDPI

Communication

SUPPLEMENTARY FIGURE

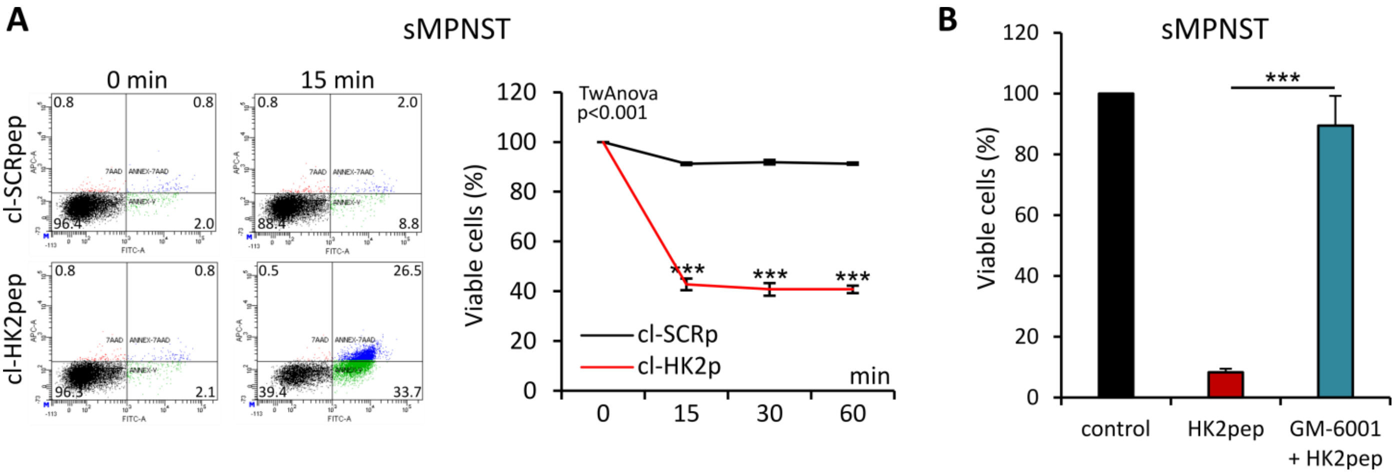

Suppl. Figure S1. (**A**) Cell death assessed by cytofluorimetry on sMPNST cells treated with 5 µM peptides (viable cells are double negative for Annexin V-FITC and 7-AAD staining). (**B**) Cell viability assessed by endpoint MTS analysis on sMPNST cells after 60 min of treatment with 10 µM HK2pep; the MMPs inhibitor GM-6001 was preincubated for 4h. cl-HK2p: cleaved HK2-targeting pep-tide; cl-SCRp: cleaved scrambled peptide; (**A**) two-way ANOVA p<0.0001, Student's t-test followed by Bonferroni post-hoc test *** p<0.001; (**B**) Student's t-test *** p<0.001.
